# Association between physical activity energy expenditure and markers of healthspan during prolonged calorie restriction in individuals without obesity: observations from the CALERIE™ phase 2 randomized controlled trial

**DOI:** 10.1186/s12966-025-01825-5

**Published:** 2025-10-07

**Authors:** James L. Dorling, Corby K. Martin, Sai Krupa Das, Susan B. Racette, Leanne M. Redman, Kim M. Huffman, Christoph Höchsmann, William E. Kraus

**Affiliations:** 1 Human Nutrition, School of Medicine, Dentistry and Nursing, College of Medical, Veterinary and Life Sciences, University of Glasgow, New Lister Building, Glasgow Royal Infirmary, 10-16 Alexandra Parade, Glasgow, G31 2ER United Kingdom; 2 Pennington Biomedical Research Center, Louisiana State University System, Baton Rouge, LA USA; 3 Jean Mayer USDA Human Nutrition Research Center on Aging, Tufts University, Boston, MA USA; 4 College of Health Solutions, Arizona State University, Phoenix, AZ USA; 5 Duke Molecular Physiology Institute, Department of Medicine, Duke University School of Medicine, Durham, NC USA; 6 Department of Health and Sport Sciences, TUM School of Medicine and Health, Technical University of Munich, Munich, Germany

**Keywords:** Caloric restriction, Energy restriction, Exercise, Longevity, Aging, Health span, Performance, Cardiovascular disease, Negative energy balance

## Abstract

**Background:**

It is unclear how physical activity energy expenditure (PAEE) influences calorie restriction (CR)-induced benefits in individuals without obesity. We examined associations between PAEE and healthspan markers and physical activity (PA) time during prolonged CR.

**Methods:**

In Comprehensive Assessment of Long-term Effects of Reducing Intake of Energy (CALERIE)™ 2, participants without obesity were randomized to 25% CR or ad libitum control. This post-hoc analysis included baseline and 24-month data from participants in both groups who demonstrated CR. PAEE was calculated from total and resting energy expenditure, measured using doubly labelled water and indirect calorimetry, respectively, and adjusted for covariates to obtain a residual value that was used as the primary exposure variable. Outcomes included grip strength, aerobic capacity, glucose, insulin, blood lipids, and self-reported PA time.

**Results:**

Overall, 136 participants (97 [71.3%] females; age: 38.6 [7.4] years; BMI: 25.3 [1.7] kg/m²) who showed CR were analyzed. A smaller decrease in PAEE was associated with improved grip strength (estimate = 0.504 [95% CI: 0.023, 0.986] kg), homeostatic model assessment of insulin resistance (estimate: -0.032 [95% CI: -0.062, -0.002]), and high-density lipoprotein-cholesterol (1.011 [95% CI: 0.356, 1.666] mg/dL; *P* ≤ 0.040). PAEE change was not associated with aerobic capacity, low-density lipoprotein-cholesterol, triglycerides, glucose, or insulin (*P* ≥ 0.053). A smaller PAEE decline was associated with more PA minutes (*P* = 0.028). For some blood lipids, change in PAEE interacted with baseline BMI class (*P* ≤ 0.029): in participants who were overweight, higher PAEE was associated with lower triglyceride and triglyceride: high-density lipoprotein-cholesterol ratio (*P* ≤ 0.022), whereas in participants who were normal weight, it was related to increased total-cholesterol (*P* = 0.006).

**Conclusion:**

A smaller reduction in PAEE during CR was associated with small improvements in several healthspan markers and greater PA time. Maintaining PAEE during CR may enhance healthspan in individuals without obesity.

**Trial registration:**

clinicaltrials.gov registration (NCT00427193).

**Supplementary Information:**

The online version contains supplementary material available at 10.1186/s12966-025-01825-5.

## Introduction

An aging population presents significant challenges for societies, including the rising prevalence of functional decline and metabolic diseases [1]. Calorie restriction (CR) is a dietary regimen that is low in calories but does not lead to malnutrition [2]. In several non-human species, CR extends life span [3], while humans who voluntarily adopt CR may improve markers associated with healthspan, which is defined as the period of life spent in good health, free from the chronic diseases and disabilities [4–6]. The Comprehensive Assessment of Long-term Effects of Reducing Intake of Energy (CALERIE)™ phase 2 study showed that two years of CR, which targeted a 25% decrease in energy intake, induced benefits in healthy humans without obesity [1], including improvements in blood lipids and decreased insulin resistance [7]. Additionally, CR improved maximal oxygen uptake (V̇O_2max_), which is related to mortality [8], and did not decrease strength relative to control [9]. This indicates that CR in healthy humans has the potential to reduce the societal burden of aging by enhancing healthspan and lowering the risk of age-related diseases.

It is unclear how physical activity-related energy expenditure (PAEE), defined as total daily energy expenditure (TDEE) minus resting metabolic rate (RMR) and the thermic effect of food, affects markers of healthspan during CR in healthy people without obesity. In CALERIE™ phase 1, aerobic exercise improved fasting insulin and blood lipids when combined with CR over 6 months, with no differences compared to CR alone [10, 11]. Research, however, is needed to test the association between PAEE and markers of healthspan during prolonged CR. Das and colleagues showed that a smaller decrease in PAEE was related to increased fat-free mass during two years of CR [12], but other areas of study are necessary. First, observations are needed to determine whether PAEE is positively associated with measures related to muscle quality and healthspan, namely strength, aerobic capacity, and cardiometabolic disease risk markers. Second, to identify targetable strategies and behaviors that alter CR-induced benefits, investigations are needed to examine whether PAEE is related to the amount of physical activity (PA) during CR. These analyses are critical as PAEE can be affected by weight loss [13] and consequently may not be directly related to PA time during CR. Third, analyses are required to ascertain whether weight status moderates the association between PAEE and healthspan markers, because CR and PA could trigger distinct health responses in individuals who are normal weight and overweight [14]. Together, such investigations will help determine whether PAEE could be modified to augment the benefits of prolonged CR and further decrease long-term disease risk in individuals without obesity who exhibit healthy metabolic parameters at baseline.

The primary aim of this post-hoc analysis was to explore the association between PAEE and markers of healthspan, namely strength, aerobic capacity, and cardiometabolic disease risk markers, during prolonged CR in individuals without obesity. The secondary aim was to examine the association between PAEE and minutes of PA during CR. The tertiary aim was to examine if weight status moderates the relationship between PAEE and healthspan markers and minutes of PA.

## Methods

### CALERIE™ phase 2

The present is a post-hoc observational analysis of the CALERIE™ phase 2 study, which was a two-year randomized clinical trial (clinicaltrials.gov registration: NCT00427193) carried out from January 2007 to November 2012 at three locations: Pennington Biomedical Research Center in Baton Rouge, LA; Washington University School of Medicine in St. Louis, MO; and Tufts University in Boston, MA. The Duke Clinical Research Institute, NC, coordinated the study [1, 15]. The protocol received approval from the institutional review boards at each site and the coordinating center, and participants gave written informed consent.

### Participants

Details of the CALERIE™ 2 trial recruitment, screening, and exclusion criteria are provided elsewhere [15–17]. In summary, 220 healthy individuals with a body mass index (BMI) of 22.0 to < 28.0 kg/m², aged 21–50 years, were randomized. Participants were ineligible if they had significant medical conditions and if they engaged in PA for 30 min for 5 or more days a week. This threshold of PA was chosen because of an anticipated intentional decrease in regular PA from individuals with high habitual activity patterns, which may confound results of the CALERIE™ 2 trial primary analysis [16].

### Study design

Participants underwent baseline testing before randomization. This included outcome assessments and habitual energy intake to establish a 25% CR target. Participants were randomized to either a CR group or an ad libitum (AL) control group for 2 years, with randomization stratified by study site, sex, and BMI; BMI was categorized into normal weight and overweight. Participants were assigned in a 2:1 ratio favoring CR within each stratum. A permuted block randomization technique was used to randomize participants by a member of the research team; treatment assignment was conducted using a telephone-based voice-response system [15].

Details of the CR intervention are available elsewhere [17]. Briefly, the intervention aimed for an immediate 25% reduction in energy intake. This was determined and monitored using the intake-balance method with doubly labelled water (DLW) measures and changes in body composition [15], which were measured using dual-energy X-ray absorptiometry (Hologic 4500 A, Delphi W, or Discovery). Further, a mathematical model was devised and guided individual participant weight change [17]. The CR group received counselling sessions led by interventionists covering strategies to support CR adherence. There were 12 group sessions delivered in the first 26 weeks and a monthly group session from weeks 27 to 104. The AL control group had quarterly sessions with investigators but did not receive any counselling. Both groups were provided with multivitamin and calcium supplements, and neither group was prescribed PA. Participants were not blinded to their intervention group, although evaluation staff were.

### Measurements

#### Physical activity energy expenditure (PAEE)

Energy expended in physical activity was estimated using assessments of TDEE and RMR. TDEE was assessed by DLW. For each DLW assessment, two baseline urine samples were collected before participants consumed a cocktail containing 0.1 g of ²H₂O (99.98% atom ²H) and 0.16 g of 100% ¹⁸O per kilogram of body weight. After ingesting the DLW, participants supplied a complete first urine void approximately 1–3 h post-ingestion, followed by six timed urine collections: two samples around 4.5 and 6 h after dosing, two on day 7 after ingestion, and two on day 14 after ingestion. Hydrogen and oxygen isotope enrichments were measured using gas-isotope-ratio mass spectrometry using validated protocols [18, 19]. The carbon dioxide production rate was calculated from the fractional turnover rates of ²H (k_H_) and ¹⁸O (k_O_) [20]. Consistent with the primary outcome paper [15], TDEE was then estimated based on the energy equivalent of a liter of CO2 to be 3.815/respiratory quotient (RQ) + 1.2321. The RQ was determined individually based on food diaries and changes in body composition; more specifically, macronutrient intakes from food diaries collected were scaled to predicted energy intake (from sex, age, fat mass, and fat-free mass), and daily changes in body stores were derived from regressions of daily home and clinic weights [15]. The resulting substrate balance was used to compute a participant-specific RQ, which was applied in the DLW calculation of total energy expenditure [15]. Indirect calorimetry was utilized to estimate RMR using a calibrated Vista-MX metabolic cart (Vacumed, Ventura, CA). PAEE was calculated using the formula: PAEE = TDEE*0.9 – RMR. TDEE was multiplied by 0.9 before subtraction of RMR because we estimated that 10% of TDEE occurs due to the thermic effect of food [9]. Similar to previous studies [11], PAEE residuals were then calculated from baseline regression models that included age, sex, fat mass, and fat-free mass as covariates. Thus, PAEE residuals were considered PAEE values adjusted for known determinants and were the primary exposure variable in this analysis, given the strong positive link between body mass and PAEE [21]. Physical activity level (PAL) was calculated using the formula: TDEE/RMR. We also used PAL as an exposure variable because, despite issues of using a ratio with a nonzero intercept [22], it is favored by some researchers [23].

#### Outcome measures

This analysis uses outcomes assessed at baseline and month 24 by evaluation staff. Grip strength was assessed using a Jamar dynamometer (Asimow Engineering Company, Los Angeles, CA). The right and left hands were tested alternately three times, with the peak recorded from each test. We calculated overall grip strength as the mean of the peak grip strength values from the left and right hands. Absolute and relative V̇O_2max_ and treadmill exercise time were determined using the Cornell treadmill test for those who met the V̇O_2max_ criteria [9]. Fasting plasma samples were analyzed for cardiometabolic disease risk markers. Total cholesterol (total-C) and triglycerides (TG) were determined using automated enzymatic commercial kits (Miles-Technicon, Tarrytown, NY, USA). High-density lipoprotein cholesterol (HDL-C) was measured by dextran sulfate (50000 MW) and low-density lipoprotein cholesterol (LDL-C) was calculated using the Friedewald Equation [24]. Plasma glucose and insulin was measured by the glucose oxidase method (YSI Instruments, Fullerton, CA, USA) and chemiluminescent immunoassay (Elecsys, Roche Diagnostics, Indianapolis, IN), respectively, and the homeostasis model assessment for insulin resistance (HOMA-IR) was calculated (fasting glucose [mmol/L] × fasting insulin [µIU/mL]/22∙5) [25]. Further, we calculated the total: HDL-C ratio and the TG: HDL ratio, given its association with cardiometabolic disease risk [26, 27]. The Stanford 7-day Physical Activity Recall questionnaire was used to estimate average self-reported minutes/day of PA [28, 29].

### Statistical analysis

Our main aims were to examine the association between change in PAEE residual and change in healthspan markers and minutes of PA during prolonged CR. Hence, the change in the PAEE residual (baseline to month 24) was the exposure variable and change in markers of healthspan and minutes of PA (baseline to month 24) were outcome variables. Completers who demonstrated CR per the intake-balance method at month 24 (percent CR > 0%) were included in the analyses, regardless of randomization group, as this method was the objective and principal indicator of CR in CALERIE™ phase 2 [15]. We also included individuals with TDEE and RMR measurements (and thus PAEE) at each timepoint.

Analyses were conducted using SPSS version 29.0 (SPSS Inc, Chicago, IL). General linear models were used to calculate change in weight, body composition, PAEE, and energy intake, with time (baseline vs. month 24) used as fixed factor. Multivariate linear regression examined the association between change in the PAEE residual and change in outcomes at month 24. Models were adjusted for site, baseline PAEE residual, BMI class (categorical variable: normal weight vs. overweight), randomization group, and baseline of the outcome variable. We performed confirmatory analyses, including: (1) the primary adjusted analysis including participants who displayed CR (percent CR > 0%) at month 12 and month 24; (2) the primary adjusted analysis with PAEE (unadjusted for known determinants) as the exposure variable; and (3) the primary adjusted analysis with PAL as the exposure variable. Furthermore, we examined if the associations between the PAEE residual and outcomes were different in individuals who were normal weight and overweight at baseline by adding BMI class (normal weight [22.0 ≤ BMI < 25.0 kg/m²] vs. overweight [25.0 ≤ BMI < 28.0 kg/m²]) as an interaction term. Significant interactions were probed by assessing the conditional relationship of PAEE residual change and change in the outcome variable for each BMI class. This was achieved by calculating estimate coefficients (95% CI) for individuals who were normal weight and overweight. The distribution of data was checked for normality and the square-root (sqrt) transformation was applied to skewed data. This was a complete case analysis, with participants removed if they had missing outcome data. A *P* value of < 0.05 was deemed statistically significant because, notwithstanding multiple testing, the project was post-hoc and thus exploratory [30].

## Results

### Participants

**Fig. 1:**
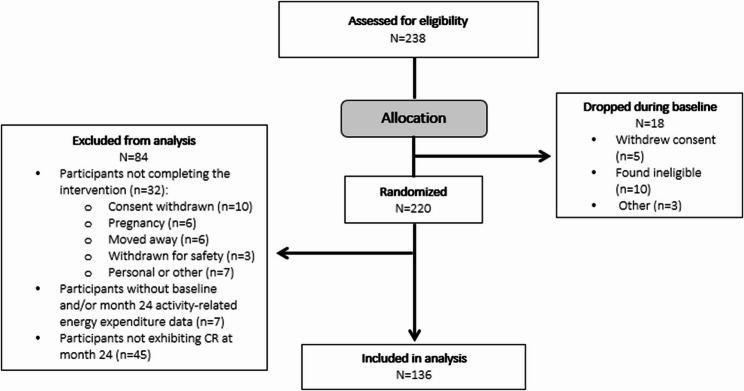
Study participant CONSORT

In total, 218 participants started the CALERIE™ 2 study. At month 24, 136 participants demonstrated CR per the DLW intake-balance method (percent CR > 0%) and had PAEE data at baseline and Follow-up at month 24; thus, these were included in the analysis (Fig. 1). Most of these participants were female (*N* = 97; 71.3%) and in the CR group (*N* = 104; 76.5%), and the mean (SD) age, weight, and BMI of this cohort was 38.6 (7.4) years, 72.6 (9.6) kg, and 25.3 (1.7) kg/m^2^, respectively (Table 1). There were 63 (46.3%) participants who were normal weight and 73 (53.7%) participants who were overweight. No between-group differences in baseline characteristics were seen (all *P* ≥ 0.207; Supplemental Table 1). Overall, participants in the analyses attained a mean (SD) CR of 11.7% (6.4%).

**Table 1:** Baseline characteristics of study participants

Variable (*N* = 136)		Values^a^
Age (year)		38.6 (7.4)
Sex		
	Male	39 (28.7)
	Female	97 (71.3)
Race		
	White	108 (79.4)
	Black	16 (11.8)
	Asian	6 (4.4)
	Other	6 (4.4)
BMI class		
	Normal weight	63 (46.3)
	Overweight	73 (53.7)
Family household income		
	$0 - $19,999	6 (4.4)
	$20,000 - $39,999	12 (8.8)
	$40,000 - $59,999	25 (18.4)
	$60,000 - $79,999	11 (8.1)
	$80,000 - $99,999	27 (19.9)
	>$100,000	55 (40.4)
Randomization group		
	Calorie restriction	104 (76.5)
	Ad libitum	32 (23.5)

^a^Continuous values are mean (± SD); categorical values are number (%)

General linear model showed weight decreased in the studied cohort (*P* < 0.001; Table 2), with weight changing by −8.4% (95% CI, −9.4, −7.3) at month 24. There was similarly a −4.3 (95% CI, −4.9, −3.7) kg and − 1.7 (−2.0, −1.4) kg change in fat mass and fat-free mass, respectively (*P* < 0.001), a reduction in measures of PAEE and PAL (*P* ≤ 0.003), and per design of the analysis, a decrease in energy intake (all *P* < 0.001; Table 2). Multiple linear regression showed that change in PAEE residual was positively related to change in energy intake after adjustments for site, baseline PAEE residual, BMI class, randomization group, and baseline energy intake (β = 0.374; *P* < 0.001).

**Table 2:** Weight, BMI, body composition, activity-related energy expenditure, and energy intake in study participants during the trial^a^

Variable (*N* = 136)	Baseline^b^	Month 24^b^	Difference^b^	*P*
Weight (kg)	72.6 (70.9, 74.2)	66.5 (64.8, 68.2)	**−6.1 (−6.9, −5.3)**	< 0.001
BMI (kg/m^2^)	25.3 (25.1, 25.6)	23.2 (22.9, 23.6)	**−2.1 (−2.4, −1.9)**	< 0.001
Fat mass (kg)	24.1 (23.3, 24.9)	19.8 (18.9, 20.7)	**−4.3 (−4.9, −3.7)**	< 0.001
Fat-free mass (kg)	48.5 (46.9, 50.0)	46.8 (45.3, 48.3)	**−1.7 (−2.0, −1.4)**	< 0.001
PAEE (kcal/day)	817 (777, 857)	693 (650, 737)	**−124 (−165, −82)**	< 0.001
PAEE residual (kcal/day)	24 (−9, 56)	−111 (−149, −74)	**−135 (−176, −95)**	< 0.001
PAL	1.76 (1.73, 1.79)	1.69 (1.66, 1.73)	**−0.06 (−0.10, −0.02)**	0.003
Energy intake (kcal/day)	2481 (2413, 2549)	2186 (2124, 2247)	**−295 (−325, −266)**	< 0.001

Bold is statistically (*P* < 0.05)

*PAEE* Physical activity energy expenditure, *BMI* Body mass index, *PAL* Physical activity level

^a^*P* values are from general linear models, with time (baseline vs. month 24) used as fixed factor. Models were complete case analysis, with participants removed if they had missing outcome data

^b^Values are means (95% CI) generated from general linear model

### Association between PAEE and outcomes

Multivariate linear regression results are displayed in Table 3. A smaller decrease (or relative increase) in PAEE residual was not associated with change in absolute V̇O_2max_ (estimate, −0.0112 [95% CI, −0.0361, 0.0137] L/min), relative V̇O_2max_ (estimate, −0.027 [95% CI, −0.445, 0.391] mL/kg/min) or change in treadmill exercise time (estimate, −0.014 [95% CI, −0.043, 0.015] sqrt min); yet a smaller reduction in the PAEE residual was associated with an increase in grip strength (*P* = 0.040). Specifically, a 100 kcal/day attenuated decline in the PAEE residual was associated with a 0.504 (0.023, 0.986) kg rise in grip strength. Further, following adjustment, an attenuated decline in the PAEE residual was associated with improvements in HOMA-IR (estimate, −0.032 [95% CI, −0.062, −0.002]; *P* = 0.036), HDL-C (estimate, 1.011 [95% CI, 0.356, 1.666] mg/dL; *P* = 0.003), and the TG: HDL ratio (estimate, −0.026 [95% CI, −0.051, −0.001] sqrt; *P* = 0.043). A smaller decrease in the PAEE residual was not, however, related to other cardiometabolic disease risk markers (*P* ≥ 0.053).

A smaller decline in the PAEE residual was associated with minutes of PA, with each 100 kcal/day attenuated reduction in the PAEE residual associated with a 0.250 (95% CI, 0.028, 0.472) sqrt min/day increase in time engaged in PA (*P* = 0.028). Analyses including all participants who completed the trial (regardless of treatment) showed a broadly similar pattern (data not shown).

**Table 3:** Association between month 24 PAEE residual change and change in outcomes during CR^a^

	Mean ± SD change	Crude model			Adjusted model^d^		
		Estimate^b^	β^c^	*P*	Estimate^b^	β^c^	*P*
Strength and aerobic capacity							
Grip strength (kg)^e^	−1.92 ± 6.85	**0.769 (0.293, 1.246)**	0.268	0.002	**0.504 (0.023, 0.986)**	0.176	0.040
V̇O_2max_ (L/min)^f^	−0.15 ± 0.24	−0.0002 (−0.0219, 0.0215)	−0.002	0.986	−0.0112 (−0.0361, 0.0137)	−0.108	0.375
V̇O_2max_ (mL/kg/min)^f^	0.98 ± 4.22	0.058 (−0.322, 0.439)	0.032	0.761	−0.027 (−0.445, 0.391)	−0.015	0.898
Treadmill exercise time (sqrt min)^f^	0.14 ± 0.30	−0.023 (−0.049, 0.004)	−0.174	0.097	−0.014 (−0.043, 0.015)	−0.111	0.327
Cardiometabolic disease risk markers							
Total-C (mg/dL)^g^	−4.58 ± 24.39	1.281 (−0.461, 3.022)	0.125	0.148	1.553 (−0.279, 3.384)	0.152	0.096
HDL-C (mg/dL)^g^	4.02 ± 8.14	**0.955 (0.393, 1.518)**	0.280	0.001	**1.011 (0.356, 1.666)**	0.296	0.003
LDL-C (mg/dL)^g^	−5.04 ± 19.75	0.651 (−0.767, 2.068)	0.078	0.366	0.885 (−0.631, 2.401)	0.107	0.250
TG (sqrt mg/dL)^g^	−0.87 ± 1.88	−0.062 (−0.197, 0.072)	−0.079	0.362	−0.070 (−0.212, 0.072)	−0.089	0.329
Total: HDL-C ratio^g^	−0.38 ± 0.64	−0.041 (−0.087, 0.004)	−0.153	0.076	−0.035 (−0.080, 0.009)	−0.130	0.122
TG: HDL ratio (sqrt)^g^	−0.18 ± 0.33	**−0.026(−0.050, −0.003)**	−0.189	0.028	**−0.026 (−0.051, −0.001)**	−0.186	0.043
Fasting glucose (mg/dL)^g^	−0.26 ± 4.71	−0.214 (−0.551, 0.123)	−0.108	0.212	−0.201 (−0.523, 0.121)	−0.102	0.219
Fasting insulin (µIU/mL)^g^	−1.17 ± 2.29	−0.038 (−0.202, 0.127)	−0.039	0.649	−0.138 (−0.279, 0.002)	−0.144	0.053
HOMA-IR^g^	−0.24 ± 0.50	−0.013 (−0.048, 0.023)	−0.061	0.482	**−0.032 (−0.062, −0.002)**	−0.155	0.036
PA minutes							
PA minutes/day (sqrt min/day)	−1.13 ± 3.05	**0.313 (0.102, 0.525)**	0.245	0.004	**0.250 (0.028, 0.472)**	0.196	0.028

Bold is statistically significant (*P* < 0.05)

*CR* Calorie restriction, *HDL-C* High-density lipoprotein cholesterol, *HOMA-IR* Homeostasis model assessment for insulin resistance, *LDL-C* Low-density lipoprotein cholesterol, *PA* Physical activity, *PAEE* Physical activity energy expenditure, *SD* Standard deviation, *TG* Triglycerides, *Total-C* Total cholesterol, *V̇O*_2max_, Maximal oxygen uptake

^a^*P* values are from multivariate linear regression of 136 participants who demonstrated CR. Regressions were complete case analysis, with participants removed if they had missing outcome data

^b^Estimates represent the change in outcome (95% CI) per 100 kcal/day increase in PAEE residual

^c^β represents the expected change in the outcome (in standard deviation units) for a one standard deviation increase in PAEE residual

^d^Model adjusted for site, BMI class, baseline PAEE residual, randomization group, and baseline of respective outcome

^e^Data were missing for 2 participants

^f^Data were missing for 44 participants

^g^Data were missing for 1 participant

**Fig. 2:**
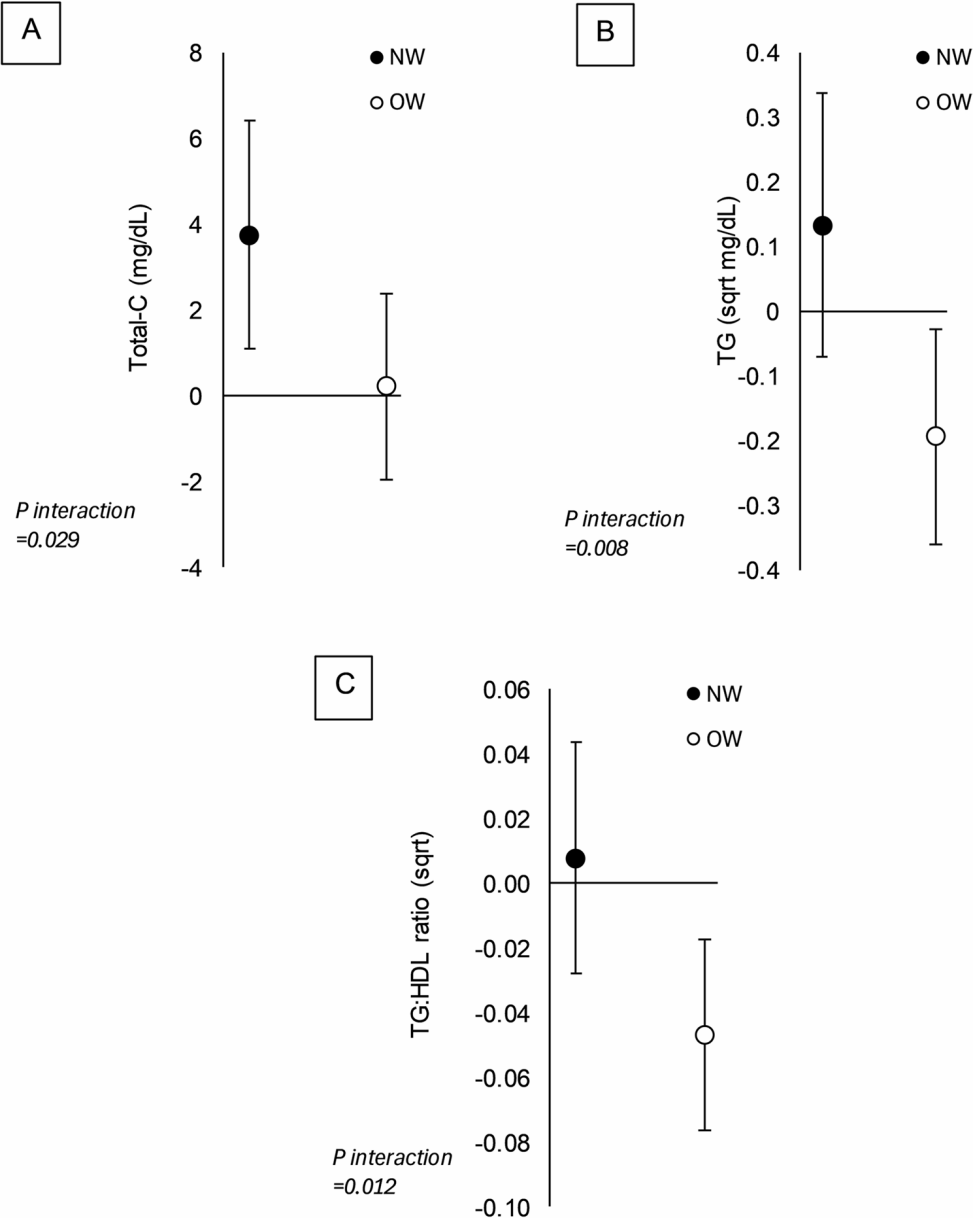
Association between change in PAEE residual and lipid markers based on weight status. Data are estimates (95% CI) representing the change in total-C (**A**), TG (**B**), and the TG: HDL ratio (**C**) per 100 kcal/day increase in PAEE residual in individuals who are normal weight (NW) and overweight (OW). Abbreviations: BMI, body mass index; HDL, high-density lipoprotein; TG, triglycerides; total-C, total cholesterol. Data missing for 1 participant

Pre-intervention BMI class moderated the association between PAEE residual change and change in total-C, TG, and the TG: HDL ratio (all interaction term *P* ≤ 0.029; Fig. 2). An attenuated decline in the PAEE residual was only associated with an increase in total-C in individuals who were normal weight (*P* = 0.006) but not overweight (*P* = 0.848), whereas an attenuated reduction in the PAEE residual was associated with a decrease in TG and the TG: HDL ratio in individuals who were overweight (all *P* ≤ 0.022) but not normal weight (all *P* ≥ 0.201; Fig. 2). The association between change in the PAEE residual and change in all other outcomes were similar in participants who were normal weight and overweight (all interaction term *P* ≥ 0.052).

As displayed in Supplemental Tables 2–4, confirmatory analyses were analogous, with similar effect sizes (standardised β values); although a smaller decrease in absolute PAEE change was associated with a significant reduction in total: HDL-C ratio and insulin (*P* ≤ 0.035; Supplemental Table 3), and an attenuated reduction in PAL was not associated with a significant reduction in the TG: HDL ratio (*P* = 0.119; Supplemental Table 4).

## Discussion

This post-hoc analysis of the CALERIE™ phase 2 trial performed in individuals without obesity examined the association between PAEE change and change in healthspan markers and PA during CR. We showed an attenuated reduction in PAEE during CR was associated with small improvements in grip strength, HOMA-IR, HDL-C, and the TG: HDL ratio; yet change in PAEE was not related to change in aerobic capacity, fasting glucose, total-C, TG, and the total: HDL-C ratio. We also showed that the change in PAEE was positively related to self-reported minutes of PA. Baseline BMI moderated some relationships, with generally favourable relationships between PAEE change and blood lipids in individuals who were overweight but not normal weight. These original findings indicate that approaches which sustain PAEE during long-term CR may enhance strength and several markers of metabolic health in healthy individuals without obesity.

Although CR induces myriad physiological and psychological benefits [1, 7, 15], a reduction in muscle function and fitness is a concern [9, 31], particularly in individuals without obesity who have lower absolute levels of fat-free mass [32]. An attenuated decline in PAEE was associated with a smaller decrease in grip strength during CR in this analysis. This relationship was small (β = 0.176), but it could be noteworthy because there is positive link between grip strength and mortality [33], and low grip strength predicts cardiovascular disease occurrence [34]. Consistent with previous evidence [35], the association between PAEE and strength occurred independent of reductions in FFM, suggesting that elevations in PAEE could enhance muscle quality during CR through neurological adaptations [36]. In contrast to strength, changes in V̇O_2max_ and treadmill exercise time were not related to change in PAEE during CR. These findings collectively indicate that methods which maintain PAEE during CR may be more beneficial for improving strength but not aerobic capacity in individuals without obesity. It is possible that CR-induced improvements in mitochondrial function and skeletal muscle oxidative efficiency [37] constrained any increases in aerobic capacity that might otherwise have been associated with PAEE, although variations in measurement sensitivity could equally play a role.

Improvements in metabolic health have been linked to increased PAEE [38]. This analysis extends these findings by showing that an attenuated reduction in PAEE during CR was related to a decrease in HOMA-IR. Reductions in HOMA-IR were seen in the CR group in CALERIE phase 2 [7]; thus, notwithstanding the small standardised beta (β = −0.155), it is notable that PAEE was related to improvements in insulin resistance in this cohort who exhibited normal HOMA-IR (1.13) at baseline. Indeed, preserving or increasing PAEE could enhance the CR-induced reductions in insulin resistance and decrease future risk of disease in healthy individuals without obesity, given insulin resistance predicts future risk of type 2 diabetes [39] and is linked to the development of metabolic disease [40].

An attenuated decline in PAEE during CR was related to a small increase in HDL-C and reduction in the TG: HDL ratio, albeit PAL change was not associated with the TG: HDL ratio and there were no significant associations with LDL-C. The mechanisms underlying these results are unknown, but sustaining PAEE may stimulate lipoprotein lipase as it is involved in HDL-C production and is increased by PA [41]. The relationship between PAEE change and the change in TG, total-C, and the TG: HDL ratio was moderated by pre-intervention BMI. Specifically, an attenuated reduction in PAEE was related to an increase in total-C in individuals who were normal weight; however, in individuals who were overweight, an attenuated reduction in PAEE during CR was related to reductions in TG and the TG: HDL ratio. These findings, on balance, suggest an attenuated decline in PAEE was associated with more favourable changes in blood lipids in individuals who were overweight, possibly mediated by increased PAEE-induced activity of lipoprotein lipase during CR. Though research is needed to examine the mechanisms involved and how alterations in PAEE differentially alters healthspan in individuals who are normal weight and overweight, this could potentially imply that preserving PAEE during CR exerts greater improvements in cardiovascular disease risk in individuals who are overweight.

Changes in metabolic efficiency [13], macronutrient intake [42], thermoregulation, the thermic effect of food, and arousal [23] could affect variations in PAEE during CR, highlighting the variety of factors affecting PAEE. Similarly, any improvement in insulin sensitivity, lipid metabolism, and strength induced by elevations in PAEE during CR could be the result of greater intrinsic metabolic efficiency, mitochondrial capacity, and muscle quality. It is likely, however, the amount PA also had a significant influence on PAEE. We observed that participants who self-reported an increase in minutes/day of PA during CR exhibited an attenuated decline in PAEE. These findings may speculatively imply that individuals without obesity who maintain higher levels of PAEE by increasing their time engaged in PA display relative improvements in strength and certain cardiometabolic health markers during CR. Moreover, they suggest that increasing PA time during CR could be an inexpensive strategy that decreases potential drawbacks of CR and further decreases disease risk. Our research does not allow us to infer what (if any) PA strategies are effective during CR in cohorts like CALERIE™ phase 2, but increasing moderate to vigorous activity, reducing sedentary time, or incorporating resistance training during both structured exercise and leisure-time physical activity may be approaches that help bolster improvements in strength and markers of metabolism during CR. Further work is needed to test the causal link between PA, PAEE, and markers of healthspan during CR, particularly given the observational nature of this analysis and the many factors influencing PAEE.

Our novel findings may help develop CR regimens that facilitate the prevention of functional decline and cardiometabolic diseases in metabolically healthy populations, yet we should note that the clinical implications of our results are equivocal given the small associations detected. For instance, using previous evidence [43], it can be estimated that a 0.032–0.096 reduction in HOMA-IR associated with an increase in PAEE of 100–300 kcal/day would reduce the relative risk of type 2 diabetes by approximately 1.9–5.5%. Though it is possible that enhancing PAEE during CR reduces type 2 diabetes and cardiometabolic disease risk through other mechanisms (e.g., inflammation) [44] and these relationships could confer significant benefits at the population level, this clinical significance is small for an individual and further work is needed to determine if elevations in PAEE and PA during CR exerts a significant improvement in long-term disease risk in healthy individuals. Research is likewise needed to elucidate how healthspan markers are affected by the interplay between PAEE and CR. This is in light of the positive association between PAEE change and energy intake change, which potentially implies that elevations in PAEE during may attenuate the degree of energy restriction achieved during CR regimens.

A strength of this analysis is that data were collected during a well-controlled 2-year CR trial, but there are limitations. First, while this analysis was post-hoc, research is needed to determine the causal relationships between PAEE and outcomes. Second, the sample size may have been too small to detect certain relationships. Third, PA minutes/day data were self-reported, introducing inherent limitations linked to recall and desirability bias [45], which may explain the small (albeit significant) association between PAEE residual change and PA change. This means objective measures like accelerometery should be used to replicate our findings. Finally, food diaries were used to estimate RQ for calculating TDEE, potentially adding error to estimates of PAEE, which is common when estimating PAEE and PAL [21, 23]. Nonetheless, the use of DLW remains a strength because it is the gold standard assessment of free-living TDEE [46], and the resources used in CALERIE™ phase 2 are exceptional.

To conclude, in individuals without obesity, a smaller reduction in PAEE during a prolonged CR regimen was associated with small yet significant improvements in grip strength, insulin resistance, HDL-C, and minutes of PA. Although work is needed to establish the clinical relevance of our findings and pinpoint approaches that optimize the benefits of CR, our findings may indicate that strategies which sustain PAEE, such as PA, could improve several indicators of healthspan in healthy individuals who adopt prolonged CR, potentially assisting the prevention of functional decline and cardiometabolic diseases.

## Supplementary Information

Supplementary Material 1.

## Data Availability

Data and a data dictionary from this study can be freely downloaded via the CALERIE™ website ( [http://calerie.duke.edu ](http:/calerie.duke.edu ) ). The corresponding author holds the analytical code and is willing to share on request.

## Abbreviations

AL: Ad libitum
BMI: Body mass index
CALERIE: Comprehensive Assessment of Long-term Effects of Reducing Intake of Energy
CR: Calorie restriction
DLW: Doubly labelled water
HDL-C: High-density lipoprotein cholesterol
HOMA-IR: Homeostasis model assessment for insulin resistance
LDL-C: Low-density lipoprotein cholesterol
PA: Physical activity
PAEE: Physical activity energy expenditure
PAL: Physical activity level
RMR: Resting metabolic rate
RQ: Respiratory quotient
sqrt: square-root
total-C: Total cholesterol
TDEE: Total daily energy expenditure
TG: Triglycerides
V̇O2max: Maximal oxygen uptake

## References

[1] Dorling JL, van Vliet S, Huffman KM, Kraus WE, Bhapkar M, Pieper CF, et al. Effects of caloric restriction on human physiological, psychological, and behavioral outcomes: highlights from CALERIE phase 2. Nutr Rev. 2021;79:98–113. 32940695 10.1093/nutrit/nuaa085 PMC7727025

[2] Redman LM, Ravussin E. Caloric restriction in humans: impact on physiological, psychological, and behavioral outcomes. Antioxid Redox Signal. 2011;14:275–87. 20518700 10.1089/ars.2010.3253 PMC3014770

[3] Heilbronn LK, Ravussin E. Calorie restriction and aging: review of the literature and implications for studies in humans. Am J Clin Nutr. 2003;78:361–9. 12936916 10.1093/ajcn/78.3.361

[4] Fontana L, Meyer TE, Klein S, Holloszy JO. Long-term calorie restriction is highly effective in reducing the risk for atherosclerosis in humans. Proc Natl Acad Sci U S A. 2004;101:6659–63. 15096581 10.1073/pnas.0308291101 PMC404101

[5] Fontana L, Weiss EP, Villareal DT, Klein S, Holloszy JO. Long-term effects of calorie or protein restriction on serum IGF-1 and IGFBP-3 concentration in humans. Aging Cell. 2008;7:681–7. 18843793 10.1111/j.1474-9726.2008.00417.x PMC2673798

[6] Kaeberlein M. How healthy is the healthspan concept? Geroscience. 2018;40:361–4. 30084059 10.1007/s11357-018-0036-9 PMC6136295

[7] Kraus WE, Bhapkar M, Huffman KM, Pieper CF, Krupa Das S, Redman LM, et al. 2 years of calorie restriction and cardiometabolic risk (CALERIE): exploratory outcomes of a multicentre, phase 2, randomised controlled trial. Lancet Diabetes Endocrinol. 2019;7:673–83. 10.1016/S2213-8587(19)30151-2. 31303390 10.1016/S2213-8587(19)30151-2 PMC6707879

[8] Ross R, Blair SN, Arena R, Church TS, Després JP, Franklin BA, et al. Importance of assessing cardiorespiratory fitness in clinical practice: a case for fitness as a clinical vital sign: a scientific statement from the American Heart Association. Circulation. 2016;134:e653-99. 27881567 10.1161/CIR.0000000000000461

[9] Racette SB, Rochon J, Uhrich ML, Villareal DT, Das SK, Fontana L, et al. Effects of two years of calorie restriction on aerobic capacity and muscle strength. Med Sci Sports Exerc. 2017;49:2240–9. 29045325 10.1249/MSS.0000000000001353 PMC5647115

[10] Heilbronn LK, De Jonge L, Frisard MI, DeLany JP, Larson-Meyer DE, Rood J, et al. Effect of 6-month calorie restriction on biomarkers of longevity, metabolic adaptation, and oxidative stress in overweight individuals: a randomized controlled trial. J Am Med Assoc. 2006;295:1539–48. 10.1001/jama.295.13.1539 PMC2692623 16595757

[11] Redman LM, Heilbronn LK, Martin CK, de Jonge L, Williamson DA, Delany JP, et al. Metabolic and behavioral compensations in response to caloric restriction: implications for the maintenance of weight loss. PLoS ONE. 2009;4:e4377. 19198647 10.1371/journal.pone.0004377 PMC2634841

[12] Das S, Roberts S, Bhapkar M, Villareal D, Fontana L, Martin C, et al. Body-composition changes in the comprehensive assessment of long-term effects of reducing intake of energy (CALERIE)-2 study: a 2-y randomized controlled trial of calorie restriction in nonobese humans. Am J Clin Nutr. 2017;105:913–27. 28228420 10.3945/ajcn.116.137232 PMC5366044

[13] Yamada Y, Colman RJ, Kemnitz JW, Baum ST, Anderson RM, Weindruch R, et al. Long-term calorie restriction decreases metabolic cost of movement and prevents decrease of physical activity during aging in rhesus monkeys. Exp Gerontol. 2013;48:1226–35. 23954367 10.1016/j.exger.2013.08.002 PMC3882119

[14] Gondim OS, de Camargo VTN, Gutierrez FA, Martins PF, de Passos O, Momesso MEP. Benefits of regular exercise on inflammatory and cardiovascular risk markers in normal weight, overweight and obese adults. PLoS ONE. 2015;10:e0140596. 26474157 10.1371/journal.pone.0140596 PMC4608693

[15] Ravussin E, Redman LM, Rochon J, Das SK, Fontana L, Kraus WE, et al. A 2-year randomized controlled trial of human caloric restriction: feasibility and effects on predictors of health span and longevity. J Gerontol A Biol Sci Med Sci. 2015;70:1097–104. 26187233 10.1093/gerona/glv057 PMC4841173

[16] Rochon J, Bales CW, Ravussin E, Redman LM, Holloszy JO, Racette SB, et al. Design and conduct of the CALERIE study: comprehensive assessment of the long-term effects of reducing intake of energy. The Journals of Gerontology: Series A. 2011;66:97–108. 10.1093/gerona/glq168 PMC3032519 20923909

[17] Rickman AD, Williamson DA, Martin CK, Gilhooly CH, Stein RI, Bales CW, et al. The CALERIE study: design and methods of an innovative 25% caloric restriction intervention. Contemp Clin Trials. 2011;32:874–81. 21767664 10.1016/j.cct.2011.07.002 PMC3185196

[18] Wong WW, Lee LS, Klein PD. Deuterium and oxygen-18 measurements on microliter samples of urine, plasma, saliva, and human milk. Am J Clin Nutr. 1987;45:905–13. 3578092 10.1093/ajcn/45.5.905

[19] Wong WW, Clarke LL, Llaurador M, Klein PD. A new zinc product for the reduction of water in physiological fluids to hydrogen gas for ^2^H/^1^H isotope ratio measurements. Eur J Clin Nutr. 1992;46:69–71. 1313759

[20] Racette SB, Schoeller DA, Luke AH, Shay K, Hnilicka J, Kushner RF. Relative dilution spaces of 2H- and 18O-labeled water in humans. Am J Physiol-Endocrinol Metab. 1994;267:E585-90. 10.1152/ajpendo.1994.267.4.E585 7943308

[21] Schutz Y, Weinsier RL, Hunter GR. Assessment of free-living physical activity in humans: an overview of currently available and proposed new measures. Obes Res. 2001;9:368–79. 11399784 10.1038/oby.2001.48

[22] Allison DB, Paultre F, Goran MI, Poehlman ET, Heymsfield SB. Statistical considerations regarding the use of ratios to adjust data. Int J Obes Relat Metab Disord. 1995;19:644–52. 8574275

[23] Speakman JR, Pontzer H. Quantifying physical activity energy expenditure based on doubly labelled water and basal metabolism calorimetry: what are we actually measuring? Curr Opin Clin Nutr Metab Care. 2023;26:401–8. 37522801 10.1097/MCO.0000000000000937

[24] Friedewald WT, Levy RI, Fredrickson DS. Estimation of the concentration of low-density lipoprotein cholesterol in plasma, without use of the preparative ultracentrifuge. Clin Chem. 1972;18:499–502. 4337382

[25] Matthews DR, Hosker JP, Rudenski AS, Naylor BA, Treacher DF, Turner RC. Homeostasis model assessment: insulin resistance and β-cell function from fasting plasma glucose and insulin concentrations in man. Diabetologia. 1985;28:412–9. 3899825 10.1007/BF00280883

[26] McLaughlin T, Abbasi F, Cheal K, Chu J, Lamendola C, Reaven G. Use of metabolic markers to identify overweight individuals who are insulin resistant. Ann Intern Med. 2003;139:802–9. 14623617 10.7326/0003-4819-139-10-200311180-00007

[27] Kannel WB, Vasan RS, Keyes MJ, Sullivan LM, Robins SJ. Usefulness of the triglyceride–high-density lipoprotein versus the cholesterol–high-density lipoprotein ratio for predicting insulin resistance and cardiometabolic risk (from the Framingham offspring cohort). Am J Cardiol. 2008;101:497–501. 18312765 10.1016/j.amjcard.2007.09.109 PMC3753679

[28] Blair SN, Haskell WL, Ho P, Paffenbarger RS, Vranizan KM, Farquhar JW, et al. Assessment of habitual physical activity by a seven-day recall in a community survey and controlled experiments. Am J Epidemiol. 1985;122:794–804. 3876763 10.1093/oxfordjournals.aje.a114163

[29] Sallis JF, Haskell WL, Wood PD, Fortmann SP, Rogers T, Blair SN, et al. Physical activity assessment methodology in the Five-City project. Am J Epidemiol. 1985;121:91–106. 3964995 10.1093/oxfordjournals.aje.a113987

[30] Anderson SF. Multiplicity in multiple regression: defining the issue, evaluating solutions, and integrating perspectives. Psychol Methods. 2023;28:1223–41. 35588079 10.1037/met0000457

[31] Prado CM, Phillips SM, Gonzalez MC, Heymsfield SB. Muscle matters: the effects of medically induced weight loss on skeletal muscle. Lancet Diabetes Endocrinol. 2024;12:785–7. 39265590 10.1016/S2213-8587(24)00272-9

[32] Magkos F. Is calorie restriction beneficial for normal-weight individuals? A narrative review of the effects of weight loss in the presence and absence of obesity. Nutr Rev. 2022;80:1811–25. 35190812 10.1093/nutrit/nuac006

[33] Rantanen T, Harris T, Leveille SG, Visser M, Foley D, Masaki K, et al. Muscle strength and body mass index as long-term predictors of mortality in initially healthy men. J Gerontol Biol Sci Med Sci. 2000;55:M168–73. 10.1093/gerona/55.3.m168 10795731

[34] Leong DP, Teo KK, Rangarajan S, Lopez-Jaramillo P, Avezum A, Orlandini A, et al. Prognostic value of grip strength: findings from the prospective urban rural epidemiology (PURE) study. Lancet. 2015;386:266–73. 25982160 10.1016/S0140-6736(14)62000-6

[35] Jung HN, Kim S-O, Jung CH, Lee WJ, Kim MJ, Cho YK. Preserved muscle strength despite muscle mass loss after bariatric metabolic surgery: a systematic review and meta-analysis. Obes Surg. 2023;33:3422–30. 37728838 10.1007/s11695-023-06796-9 PMC10602996

[36] Folland JP, Williams AG. The adaptations to strength training: morphological and neurological contributions to increased strength. Sports Med. 2007;37:145–68. 17241104 10.2165/00007256-200737020-00004

[37] Civitarese AE, Carling S, Heilbronn LK, Hulver MH, Ukropcova B, Deutsch WA, et al. Calorie restriction increases muscle mitochondrial biogenesis in healthy humans. PLoS Med. 2007;4:e76. 17341128 10.1371/journal.pmed.0040076 PMC1808482

[38] Karelis AD, Lavoie M-È, Messier V, Mignault D, Garrel D, Prud’homme D, et al. Relationship between the metabolic syndrome and physical activity energy expenditure: a MONET study. Appl Physiol Nutr Metab. 2008;33:309–14. 18347686 10.1139/H07-193

[39] Song Y, Manson JE, Tinker L, Howard BV, Kuller LH, Nathan L, et al. Insulin sensitivity and insulin secretion determined by homeostasis model assessment and risk of diabetes in a multiethnic cohort of women. Diabetes Care. 2007;30:1747–52. 17468352 10.2337/dc07-0358 PMC1952235

[40] Reaven GM. Role of insulin resistance in human disease. Diabetes. 1988;37:1595–607. 3056758 10.2337/diab.37.12.1595

[41] Ferguson MA, Alderson NL, Trost SG, Essig DA, Burke JR, Durstine JL. Effects of four different single exercise sessions on lipids, lipoproteins, and lipoprotein lipase. J Appl Physiol. 1998;85:1169–74. 9729596 10.1152/jappl.1998.85.3.1169

[42] Hall KD, Guo J, Chen KY, Leibel RL, Reitman ML, Rosenbaum M, et al. Methodologic considerations for measuring energy expenditure differences between diets varying in carbohydrate using the doubly labeled water method. Am J Clin Nutr. 2019;109:1328–34. 31028699 10.1093/ajcn/nqy390 PMC6499509

[43] Song Y, Manson JE, Tinker L, Howard BV, Kuller LH, Nathan L, et al. Insulin sensitivity and insulin secretion determined by homeostasis model assessment and risk of diabetes in a multiethnic cohort of women: the women’s health initiative observational study. Diabetes Care. 2007;30:1747–52. 17468352 10.2337/dc07-0358 PMC1952235

[44] Fedewa MV, Hathaway ED, Ward-Ritacco CL. Effect of exercise training on C reactive protein: a systematic review and meta-analysis of randomised and non-randomised controlled trials. Br J Sports Med. 2017;51:670–6. 27445361 10.1136/bjsports-2016-095999

[45] Prince SA, Adamo KB, Hamel M, Hardt J, Connor Gorber S, Tremblay M. A comparison of direct versus self-report measures for assessing physical activity in adults: a systematic review. Int J Behav Nutr Phys Act. 2008;5:56. 18990237 10.1186/1479-5868-5-56 PMC2588639

[46] Irwin ML, Ainsworth BE, Conway JM. Estimation of energy expenditure from physical activity measures: determinants of accuracy. Obes Res. 2001;9:517–25. 11557832 10.1038/oby.2001.68

