# Supplementary material for: Association between physical activity energy expenditure and markers of healthspan during prolonged calorie restriction in individuals without obesity: observations from the CALERIE™ phase 2 randomized controlled trial
Source: Int J Behav Nutr Phys Act. 2025 Oct 7;22:124. doi: 10.1186/s12966-025-01825-5 (PMC12505589; doi:10.1186/s12966-025-01825-5)
Supplement: Supplementary file 1 — Supplementary Material 1. [file 12966_2025_1825_MOESM1_ESM.docx]

**SUPPLEMENTAL ONLINE CONTENT**

Dorling et al. Association between physical activity energy expenditure and markers of healthspan during prolonged calorie restriction in individuals without obesity: observations from the CALERIE™ phase 2 randomized controlled trial.

**Supplemental Table 1.** Baseline characteristics of study participants in the calorie restriction and ad libitum groups.

| Variable |  | Calorie restriction  (N = 104) ^a^ | Ad libitum  (N = 32) ^a^ | *P*^b^ |
| --- | --- | --- | --- | --- |
| Age (year) |  | 38.8 (7.4) | 37.9 (7.3) | 0.550 |
| Sex |  |  |  | 0.937 |
|  | Male | 30 (28.8) | 9 (28.1) |  |
|  | Female | 74 (71.2) | 23 (71.9) |  |
| Race |  |  |  | 0.874 |
|  | White | 82 (78.8) | 26 (81.3) |  |
|  | Black | 13 (12.5) | 3 (9.4) |  |
|  | Asian | 5 (4.8) | 1 (3.1) |  |
|  | Other | 4 (3.8) | 2 (6.3) |  |
| BMI class |  |  |  | 0.943 |
|  | Normal weight | 48 (46.2) | 15 (46.9) |  |
|  | Overweight | 56 (53.8) | 17 (53.1) |  |
| Family household income |  |  |  | 0.207 |
|  | $0 - $19,999 | 5 (4.8) | 1 (3.1) |  |
|  | $20,000 - $39,999 | 7 (6.7) | 5 (15.6) |  |
|  | $40,000 - $59,999 | 21 (20.2) | 4 (12.5) |  |
|  | $60,000 - $79,999 | 11 (10.6) | 0 (0.0) |  |
|  | $80,000 - $99,999 | 19 (18.3) | 8 (25.0) |  |
|  | >$100,000 | 41 (39.4) | 14 (43.8) |  |

^a^ Continuous values are mean (±SD); categorical values are number (%).

^b^*P* value for continuous variables derived from independent t-test; *P* value for categorical variables derived from chi-square test.

**Supplemental Table 2.** Association between month 24 PAEE residual change and change in outcomes during CR.^a^

|  |  | Crude model | | |  | Adjusted model^d^ | | |
| --- | --- | --- | --- | --- | --- | --- | --- | --- |
|  | | Estimate^b^ | β^c^ | *P* |  | Estimate^b^ | β^c^ | *P* |
| Strength and aerobic capacity | |  |  |  |  |  |  |  |
|  | Grip strength (kg)^e^ | **0.766 (0.287, 1.246)** | 0.267 | 0.002 |  | **0.493 (0.008, 0.978)** | 0.172 | 0.047 |
|  | V̇O_2max_ (L/min)^f^ | -0.0004 (-0.0219, 0.0212) | -0.004 | 0.973 |  | -0.0097 (-0.0348, 0.0153) | -0.096 | 0.441 |
|  | V̇O_2max_ (mL/kg/min)^f^ | 0.056 (-0.325, 0.438) | 0.031 | 0.770 |  | 0.007 (-0.409, 0.423) | 0.004 | 0.974 |
|  | Treadmill exercise time (sqrt min)^f^ | -0.023 (-0.050, 0.004) | -0.175 | 0.100 |  | -0.013 (-0.042, 0.016) | -0.101 | 0.378 |
| Cardiometabolic disease risk markers | |  |  |  |  |  |  |  |
|  | Total-C (mg/dL)^g^ | 1.302 (-0.446, 3.051) | 0.128 | 0.143 |  | 1.504 (-0.338, 3.345) | 0.147 | 0.109 |
|  | HDL-C (mg/dL)^g^ | **0.970 (0.414, 1.525)** | 0.289 | <0.001 |  | **0.970 (0.314, 1.627)** | 0.289 | 0.004 |
|  | LDL-C (mg/dL)^g^ | 0.663 (-0.763, 2.088) | 0.080 | 0.359 |  | 0.835 (-0.688, 2.359) | 0.101 | 0.280 |
|  | TG (sqrt mg/dL)^g^ | -0.064 (-0.199, 0.072) | -0.081 | 0.354 |  | -0.067 (-0.211, 0.076) | -0.086 | 0.355 |
|  | Total:HDL-C ratio^g^ | -0.042 (-0.088, 0.004) | -0.155 | 0.074 |  | -0.035 (-0.081, 0.010) | -0.132 | 0.122 |
|  | TG:HDL ratio (sqrt)^g^ | **-0.027 (-0.050, -0.003)** | -0.193 | 0.026 |  | -0.025 (-0.050, 0.000) | -0.182 | 0.050 |
|  | Fasting glucose (mg/dL)^g^ | -0.213 (-0.552, 0.127) | -0.108 | 0.217 |  | -0.198 (-0.523, 0.127) | -0.100 | 0.230 |
|  | Fasting insulin (μIU/mL)^g^ | -0.040 (-0.205, 0.126) | -0.041 | 0.636 |  | **-0.142 (-0.284, 0.000)** | -0.148 | 0.050 |
|  | HOMA-IR^g^ | -0.013 (-0.049, 0.023) | -0.063 | 0.472 |  | **-0.033 (-0.063, -0.003)** | -0.159 | 0.034 |
| PA minutes | |  |  |  |  |  |  |  |
|  | PA minutes/day (sqrt min) | **0.314 (0.102, 0.526)** | 0.247 | 0.004 |  | **0.258 (0.036, 0.480)** | 0.203 | 0.023 |

Abbreviations: CR, calorie restriction; HDL-C, high-density lipoprotein cholesterol; HOMA-IR, homeostasis model assessment for insulin resistance; LDL-C, low-density lipoprotein cholesterol; PA, physical activity; PAEE, physical activity energy expenditure; TG, triglycerides; total-C, total cholesterol; V̇O2max, maximal oxygen uptake.

^a^*P* values are from multivariate linear regression of 134 participants who demonstrated CR at month 12 and month 24. Regressions were complete case analysis, with participants removed if they had missing outcome data.

^b^Estimates represent the change in outcome (95% CI) per 100 kcal/day increase in PAEE residual.

^c^β represents the expected change in the outcome (in standard deviation units) for a one standard deviation increase in PAEE residual.

^d^Model adjusted for site, BMI class, baseline PAEE residual, randomization group, and baseline of respective outcome.

^e^Data were missing for 2 participants.

^f^Data were missing for 44 participants.

^g^Data were missing for 1 participant.

Bold is statistically significant (*P* < 0.05).

**Supplemental Table 3.** Association between month 24 PAEE change and change in outcomes during CR.^a^

|  |  | Crude model | | |  | Adjusted model^d^ | | |
| --- | --- | --- | --- | --- | --- | --- | --- | --- |
|  | | Estimate^b^ | β^c^ | *P* |  | Estimate^b^ | β^c^ | *P* |
| Strength and aerobic capacity | |  |  |  |  |  |  |  |
|  | Grip strength (kg)^e^ | **0.721 (0.258, 1.183)** | 0.259 | 0.002 |  | **0.782 (0.341, 1.222)** | 0.281 | <0.001 |
|  | V̇O_2max_ (L/min)^f^ | 0.002 (-0.019, 0.023) | 0.024 | 0.821 |  | -0.009 (-0.033, 0.015) | -0.090 | 0.462 |
|  | V̇O_2max_ (mL/kg/min)^f^ | 0.128 (-0.239, 0.495) | 0.073 | 0.490 |  | 0.039 (-0.364, 0.443) | 0.022 | 0.847 |
|  | Treadmill exercise time (sqrt min)^f^ | -0.018 (-0.044, 0.008) | -0.145 | 0.169 |  | -0.003 (-0.031, 0.025) | -0.027 | 0.809 |
| Cardiometabolic disease risk markers | |  |  |  |  |  |  |  |
|  | Total-C (mg/dL)^g^ | 1.309 (-0.377, 2.995) | 0.132 | 0.127 |  | 0.830 (-0.902, 2.562) | 0.084 | 0.345 |
|  | HDL-C (mg/dL)^g^ | **0.959 (0.416, 1.502)** | 0.290 | 0.001 |  | **0.942 (0.331, 1.553)** | 0.285 | 0.003 |
|  | LDL-C (mg/dL)^g^ | 0.596 (-0.778, 1.969) | 0.074 | 0.392 |  | 0.374 (-1.055, 1.803) | 0.047 | 0.606 |
|  | TG (sqrt mg/dL)^g^ | -0.046 (-0.176, 0.085) | -0.060 | 0.492 |  | -0.083 (-0.216, 0.050) | -0.109 | 0.218 |
|  | Total:HDL-C ratio^g^ | -0.039 (-0.083, 0.006) | -0.147 | 0.088 |  | **-0.045 (-0.086, -0.003)** | -0.171 | 0.035 |
|  | TG:HDL ratio (sqrt)^g^ | **-0.023 (-0.046, -0.001)** | -0.173 | 0.045 |  | **-0.027 (-0.050, -0.003)** | -0.198 | 0.025 |
|  | Fasting glucose (mg/dL)^g^ | -0.231 (-0.557, 0.095) | -0.121 | 0.163 |  | -0.109 (-0.421, 0.202) | -0.057 | 0.488 |
|  | Fasting insulin (μIU/mL)^g^ | -0.030 (-0.190, 0.129) | -0.033 | 0.706 |  | **-0.166 (-0.295, -0.037)** | -0.179 | 0.012 |
|  | HOMA-IR^g^ | -0.011 (-0.046, 0.023) | -0.056 | 0.520 |  | **-0.036 (-0.063, -0.008)** | -0.178 | 0.011 |
| PA minutes | |  |  |  |  |  |  |  |
|  | PA minutes/day (sqrt min) | **0.307 (0.102, 0.512)** | 0.248 | 0.004 |  | **0.299 (0.096, 0.503)** | 0.242 | 0.004 |

Abbreviations: CR, calorie restriction; HDL-C, high-density lipoprotein cholesterol; HOMA-IR, homeostasis model assessment for insulin resistance; LDL-C, low-density lipoprotein cholesterol; PA, physical activity; PAEE, physical activity energy expenditure; TG, triglycerides; total-C, total cholesterol; V̇O2max, maximal oxygen uptake.

^a^*P* values are from multivariate linear regression of 136 participants who demonstrated CR. Regressions were complete case analysis, with participants removed if they had missing outcome data.

^b^Estimates represent the change in outcome (95% CI) per 100 kcal/day increase in PAEE.

^c^β represents the expected change in the outcome (in standard deviation units) for a one standard deviation increase in PAEE.

^d^Model adjusted for site, BMI class, baseline PAEE, randomization group, and baseline of respective outcome.

^e^Data were missing for 2 participants.

^f^Data were missing for 44 participants.

^g^Data were missing for 1 participant.

Bold is statistically significant (*P* < 0.05).

**Supplemental Table 4.** Association between month 24 PAL change and change in outcomes during CR.^a^

|  |  | Crude model | | |  | Adjusted model^d^ | | |
| --- | --- | --- | --- | --- | --- | --- | --- | --- |
|  | | Estimate^b^ | β^c^ | *P* |  | Estimate^b^ | β^c^ | *P* |
| Strength and aerobic capacity | |  |  |  |  |  |  |  |
|  | Grip strength (kg)^e^ | **0.816 (0.355, 1.277)** | 0.292 | <0.001 |  | **0.616 (0.135, 1.096)** | 0.220 | 0.012 |
|  | V̇O_2max_ (L/min)^f^ | -0.001 (-0.022, 0.020) | -0.009 | 0.931 |  | -0.013 (-0.037, 0.010) | -0.132 | 0.273 |
|  | V̇O_2max_ (mL/kg/min)^f^ | 0.109 (-0.256, 0.473) | 0.062 | 0.554 |  | -0.065 (-0.463, 0.332) | -0.037 | 0.745 |
|  | Treadmill exercise time (sqrt min)^f^ | -0.024 (-0.049, 0.002) | -0.189 | 0.071 |  | -0.015 (-0.043, 0.013) | -0.121 | 0.283 |
| Cardiometabolic disease risk markers | |  |  |  |  |  |  |  |
|  | Total-C (mg/dL)^g^ | 1.000 (-0.705, 2.705) | 0.100 | 0.248 |  | 1.135 (-0.705, 2.974) | 0.114 | 0.224 |
|  | HDL-C (mg/dL)^g^ | **0.917 (0.367, 1.466)** | 0.275 | 0.001 |  | **0.884 (0.228, 1.539)** | 0.265 | 0.009 |
|  | LDL-C (mg/dL)^g^ | 0.322 (-1.064, 1.709) | 0.040 | 0.647 |  | 0.574 (-0.941, 2.090) | 0.071 | 0.455 |
|  | TG (sqrt mg/dL)^g^ | -0.046 (-0.178, 0.086) | -0.060 | 0.489 |  | -0.047 (-0.189, 0.095) | -0.062 | 0.511 |
|  | Total:HDL-C ratio^g^ | **-0.046 (-0.091, -0.002)** | -0.176 | 0.042 |  | -0.035 (-0.080, 0.009) | -0.135 | 0.116 |
|  | TG:HDL ratio (sqrt)^g^ | **-0.023 (-0.046, <0.001)** | -0.170 | 0.048 |  | -0.020 (-0.045, 0.005) | -0.146 | 0.119 |
|  | Fasting glucose (mg/dL)^g^ | -0.222 (-0.551, 0.107) | -0.115 | 0.184 |  | -0.160 (-0.484, 0.164) | -0.083 | 0.330 |
|  | Fasting insulin (μIU/mL)^g^ | -0.042 (-0.203, 0.118) | -0.045 | 0.602 |  | **-0.181 (-0.318, -0.043)** | -0.193 | 0.010 |
|  | HOMA-IR^g^ | -0.013 (-0.048, 0.022) | -0.064 | 0.461 |  | **-0.040 (-0.069, -0.010)** | -0.196 | 0.008 |
| PA minutes | |  |  |  |  |  |  |  |
|  | PA minutes/day (sqrt min) | **0.267 (0.059, 0.475)** | 0.214 | 0.012 |  | **0.242 (0.021, 0.462)** | 0.194 | 0.032 |

Abbreviations: CR, calorie restriction; HDL-C, high-density lipoprotein cholesterol; HOMA-IR, homeostasis model assessment for insulin resistance; LDL-C, low-density lipoprotein cholesterol; PA, physical activity; PAL, physical activity level; TG, triglycerides; total-C, total cholesterol; V̇O2max, maximal oxygen uptake.

^a^*P* values are from multivariate linear regression of 136 participants who demonstrated CR. Regressions were complete case analysis, with participants removed if they had missing outcome data.

^b^Estimates represent the change in outcome (95% CI) per 0.1 increase in PAL.

^c^β represents the expected change in the outcome (in standard deviation units) for a one standard deviation increase in PAL.

^d^Model adjusted for site, BMI class, baseline PAL, randomization group, and baseline of respective outcome.

^e^Data were missing for 2 participants.

^f^Data were missing for 44 participants.

^g^Data were missing for 1 participant.

Bold is statistically significant (*P* < 0.05).
